# Morphia and Ergot as Parturients

**Published:** 1871-08

**Authors:** Geo. H. Vance


### Correspondence.

### MORPHIA AND ERGOT AS PARTURIENTS.

In answer to the queries of Dr. Robinson, in relation to the *modus operandi* of morphia and ergot, Dr. Vance writes as follows:

1st. What the *modus operandi* of secale cornutum is.

In the first place, it is received in what is termed the exterior part of the system as per orem or per anum; then it is taken up by the veinous absorbent and conducted into the interior system or general circulation, and thence is conveyed along and taken up by the ganglionic nerves—especially the sacral plexus—or rather the hypogastric plexus, which operates immediately on the fibres of the fundus interi, exciting in them strong and persistent efforts of contractility. Although secale cornutum has different properties applied to it, one thing is evident, *i.e.*, its stimulating propensities, as exerted in the fibres of the womb, forwarding expulsion of fœtus and secundines, through the agency of the great sympathetic nerves.

2d. What is the *modus operandi* of sulphate morphia upon the parturient patient?

Sulphate morphia is received into the system not unlike the remedy spoken of, excepting hypodermically; but upon the uterine fibres it seems to impart two co-operating effects: the first of which is a stimulant, operating through the sympathetic system, producing contractility of the fibres not unlike secale cornutum, favoring the expulsive efforts; and, secondly, that of an anodyne effect, through the spinal nerves, upon the circular fibres of the os uteri, thereby admirably facilitating dilatation, and, thankfully to patient and attendant, accelerating the item, *deliverance.*

GEO. H. VANCE.

